# Split Nitrogen Application Improves Wheat Baking Quality by Influencing Protein Composition Rather Than Concentration

**DOI:** 10.3389/fpls.2016.00738

**Published:** 2016-06-01

**Authors:** Cheng Xue, Gunda Schulte auf’m Erley, Anne Rossmann, Ramona Schuster, Peter Koehler, Karl-Hermann Mühling

**Affiliations:** ^1^Faculty of Agricultural and Nutritional Sciences, Institute of Plant Nutrition and Soil Science, Kiel UniversityKiel, Germany; ^2^College of Resources and Environment Science, Agricultural University of HebeiBaoding, China; ^3^Deutsche Forschungsanstalt für Lebensmittelchemie, Leibniz InstitutFreising, Germany

**Keywords:** late nitrogen application, gliadins, glutenins, gluten subunits, bread loaf volume

## Abstract

The use of late nitrogen (N) fertilization (N application at late growth stages of wheat, e.g., booting, heading or anthesis) to improve baking quality of wheat has been questioned. Although it increases protein concentration, the beneficial effect on baking quality (bread loaf volume) needs to be clearly understood. Two pot experiments were conducted aiming to evaluate whether late N is effective under controlled conditions and if these effects result from increased N rate or N splitting. Late N fertilizers were applied either as additional N or split from the basal N at late boot stage or heading in the form of nitrate-N or urea. Results showed that late N fertilization improved loaf volume of wheat flour by increasing grain protein concentration and altering its composition. Increasing N rate mainly enhanced grain protein quantitatively. However, N splitting changed grain protein composition by enhancing the percentages of gliadins and glutenins as well as certain high molecular weight glutenin subunits (HMW-GS), which led to an improved baking quality of wheat flour. The late N effects were greater when applied as nitrate-N than urea. The proportions of glutenin and x-type HMW-GS were more important than the overall protein concentration in determining baking quality. N splitting is more effective in improving wheat quality than the increase in the N rate by late N, which offers the potential to cut down N fertilization rates in wheat production systems.

## Introduction

Wheat, one of the main food crops, is unique for the viscoelastic properties of its dough which could be processed mainly into bread for hard wheat. Two crucial determinants of bread-making quality are the grain protein concentration and protein composition, including the amounts and ratios of gluten protein fractions as well as their subunits (Weegels et al., 1996; Zhang et al., 2009). Gluten proteins, consisting of gliadins and glutenins, play an important role in the bread-making quality of wheat flour as gliadins mainly contribute to dough viscosity and extensibility, while glutenins to dough strength and elasticity (Wieser, 2007).

Wheat bread-making quality is highly affected by both genetic and environmental factors. Within environmental factors, fertilization, especially nitrogen (N) fertilization plays an important role and can be easily adjusted comparing to climatic and irrigation conditions. Numerous studies demonstrated that wheat bread-making quality was improved with higher N fertilization rate mainly through the increase in grain protein concentration, as well as gliadin to glutenin and high molecular weight glutenin subunits (HMW-GS) to low molecular weight glutenin subunits (LMW-GS) ratios (Jia et al., 1996; Wieser and Seilmeier, 1998; Garrido-Lestache et al., 2004; Fuertes-Mendizábal et al., 2010; Zörb et al., 2010).

Therefore, late N fertilization (application of additional N at heading or anthesis) is widely recognized as useful for enhancing grain protein concentration and is practically conducted. Many studies have reported improved bread-making quality of wheat flour after late N fertilization (Wieser and Seilmeier, 1998; Blandino et al., 2015). This effect may result not only from increased total N fertilization rate but also from split N application. The split N application (splitting of the same N rate distributed in several applications at different growth stages) also influences the wheat flour quality since the delayed N application predominantly benefits protein build-up over starch in the grain and prolongs the duration of grain-filling (Sowers et al., 1994). However, the results of split N application on wheat quality varied among studies (Garrido-Lestache et al., 2004; Fuertes-Mendizábal et al., 2010; Schulz et al., 2015). The positive effects may be attributed mainly to the N rate effect since the fertilizer N use efficiency can be enhanced by split N application under appropriate environmental conditions, which resulted in higher plant N uptake (Hamid, 1972; Ercoli et al., 2013). On the contrary, the delayed application of fertilizer N might not be taken up efficiently by plants under unfavorable field conditions, thus no such positive effect was achieved from split N application. Similarly, it was also shown that the effects of late N fertilization varied between different environmental conditions such as locations and total N fertilization rate (Jia et al., 1996). Therefore, it is hypothesized that the effects of late N fertilization on wheat grain protein concentration and composition were actually due to N fertilization rate and not to split N application. If this is true, the late N fertilizer could be combined with early applications, thus simplifying the N fertilization management. Besides, taking into account the practical problems and extra costs of applying a late N dose, further investigations are needed on whether a late N application is necessary. To clarify the split N application effect and to distinguish it from the N rate effect, the plant N uptake and recovery of the late fertilizer N should be considered under controlled conditions. Unfortunately, limited studies aimed at this issue are available.

In order to avoid N losses and contamination of underground water and air through leaching and gaseous losses, the accumulation of large amount of soil-nitrate should be avoided. Unlike nitrate-N, ammonium-N cannot be stored in plant cells after uptake by plants. Therefore, it is hypothesized that the process of amino acids synthesis from ammonium-fed wheat would be enhanced, thus promoting protein accumulation in the final grain. Fuertes-Mendizábal et al. (2013) reported that ammonium as sole N source combined with split N application improved grain quality in wheat compared to nitrate-N source. Nevertheless, the differences between N forms as late N application on wheat grain quality still need further investigation.

Bread-making quality of cultivars differing in protein concentration or quality (such as HMW-GS composition) may respond differently to variations in protein concentration (Weegels et al., 1996). For cultivars exhibiting high baking quality with relatively low protein concentration, measures to increase protein concentration might be dispensable. Therefore, to investigate the effect of late N fertilization more comprehensively, cultivars belonging to different quality groups should be considered. Moreover, the correlation between protein concentration and bread-making quality varies widely over years and cultivars (Koppel and Ingver, 2010). Therefore, a suitable baking test is needed to avoid incorrect evaluation of the bread-making quality of wheat cultivars. Unfortunately, very limited studies have used the baking test to evaluate wheat flour quality due to a lack of material or equipment.

The main objectives of this study were (i) to evaluate the effect of late N fertilization on the quantity and composition of gluten proteins and thus baking quality (loaf volume), (ii) to distinguish whether this effect resulted from higher N rate or split N application, (iii) to determine how different N forms affect the protein and baking quality when applied as late N, (iv) to determine how cultivars belonging to different quality groups respond to late N fertilization, (v) to check if protein concentration is a reliable parameter in evaluating baking quality under such conditions.

## Materials and Methods

### Experimental Design

Two winter wheat (*Triticum aestivum* L.) cultivars Tobak (W. von Borries-Eckendorf, Leopoldshöhe, Germany) and JB Asano (Saatzucht Josef Breun, Herzogenaurach, Germany), belonging to different baking quality classes according to the German Federal Office of Plant Varieties, were used in this study. Tobak (baking quality class B) had similar loaf volume but lower raw protein concentration comparing to JB Asano (baking quality class A). Besides, according to ‘Glu-1 quality score’ (Payne et al., 1987) based on HMW-GS, the ‘Glu-1 quality score’ for Tobak is five, whereas for JB Asano it is seven. An N fertilization experiment was conducted in the year 2011/2012, and was repeated in the following year 2012/2013 with modifications in total N fertilization rate and timing of late N application.

The first experiment (2011/2012) was performed in Mitscherlich pots (diameter: 21 cm, depth: 21.5 cm) containing 6 kg of soil with supplemental irrigation under natural conditions, i.e., outdoor, except during strong frost and exceptionally high rainfall. The fertilization treatments were designed as follows and summarized in **Table 1**: N1 (low N rate treatment), an N rate of 1.5 g N pot^-1^ in two doses with 1 g N pot^-1^ applied before seeding and 0.5 g N pot^-1^ at EC30 (beginning of stem elongation) (Lancashire et al., 1991); N2 (higher N rate treatment), 2 g N pot^-1^ in two doses with 1 g N pot^-1^ applied before seeding and 1 g N pot^-1^ at EC30; N3 (late nitrate treatment), 2 g N pot^-1^ applied in three doses with 1 g N pot^-1^ applied before seeding, 0.5 N pot^-1^ at EC30 and 0.5 g N pot^-1^ in the form of calcium nitrate (^15^N labeled) applied as a late N application at EC45 (late boot stage); N4 (late urea treatment), 2 g N pot^-1^ applied in three doses with 0.5 g N pot^-1^ in the form of urea (^15^N labeled) with nitrification inhibitor dicyandiamide (DCD) applied as a late N application at EC45 comparing to N3. Thus, the effects of N fertilization rate (N2 vs. N1), split N application (N3 and N4 vs. N2), late N fertilization (N3 and N4 vs. N1) and N forms (N4 vs. N3) could be determined in the present study. Besides, the recovery and harvest index of late N fertilizer could be determined by using ^15^N labeled fertilizers. The N fertilizers applied before seeding and at EC30 for all treatments were in the form of ammonium nitrate. Other nutrients including P (0.6 g pot^-1^), K (2.3 g pot^-1^), S (0.5 g pot^-1^), Mg (0.33 g pot^-1^), Ca (1.19 g pot^-1^) and the minor elements Cu (10 mg pot^-1^), Zn (15 mg pot^-1^) and Mn (30 mg pot^-1^) were applied before winter wheat seeding. The basal fertilizers NH_4_⋅NO_3_, K_2_⋅SO_4_, Mg(NO_3_)_2_⋅6H_2_O, KCl, CuSO_4_⋅5H_2_O, MnSO_4_⋅H_2_O and ZnSO_4_⋅7H_2_O were applied as liquid solution, while Ca(H_2_PO_4_)⋅2H_2_O and CaCO_3_ were applied as solids. The fertilizers were mixed into the soil for five replicates and then uniformly separated into five pots 1 week prior to seeding. For the second and third fertilization liquid fertilizer was uniformly applied onto the surface of each pot. Winter wheat was sown on 30 November 2011 and harvested on 6 August 2012. Nitrogen fertilization was conducted on 25 November 2011, 2 April 2012 and 30 May 2012, respectively. Fungi disease and insects were well controlled by spraying fungicide Capalo three times and insecticide Biscaya once during winter wheat growth.

**Table 1:** Summary of N fertilization (N rate, g N pot^-1^).

		EC 0	EC 30	EC 45		EC 51		
Treatments^a^		N rate	N rate	N rate	N type	N rate	N type	Total N rate
Experiment 1	N1 (1 + 0.5 + 0)	1	0.5	0	–	0	–	1.5
	N2 (1 + 1 + 0)	1	1	0	–	0	–	2
	N3 (1 + 0.5 + 0.5N)	1	0.5	0.5	Ca(NO_3_)_2_⋅4H_2_O	0	–	2
	N4 (1 + 0.5 + 0.5U)	1	0.5	0.5	Urea + DCD	0	–	2

Experiment 2	N5 (1 + 1 + 0)	1	1	0	–	0	–	2
	N6 (1 + 1.5 + 0)	1	1.5	0	–	0	–	2.5
	N7 (1 + 1 + 0.5N)	1	1	0	–	0.5	Ca(NO_3_)_2_⋅4H_2_O	2.5
	N8 (1 + 1 + 0.5U)	1	1	0	–	0.5	Urea + DCD	2.5

*^a^In the first experiment, N1 (1 + 0.5 + 0) represents treatment N1, with N fertilization rate and timing at 1 g N pot^-1^ before sowing, 0.5 g N pot^-1^ at EC30 (stem elongation), and 0 g N pot^-1^ at EC45 (late boot stage). The N fertilization rate and timing in treatment N2, N3, and N4 were done in the same way as N1. In the second experiment, N5 (1 + 1 + 0) represents treatment N5, with N fertilization rate and timing at 1 g N pot^-1^ before sowing, 1 g N pot^-1^ at EC30, and 0 g N pot^-1^ at EC51 (beginning of heading). The N fertilization rate and timing in treatment N6, N7, and N8 were done in the same way as N5. The expression ‘0.5N’ means that 0.5 g N pot^-1^ was given as calcium nitrate, ‘0.5U’ means that 0.5 g N pot^-1^ was given as urea plus nitrification inhibitor DCD.*

The second experiment (2012/2013) was conducted as a replicate of the first experiment with modifications. For each treatment (N5, N6, N7, and N8 comparing to N1, N2, N3, and N4, respectively), 0.5 g more N pot^-1^ was given at EC30 and the application of late N fertilizer was postponed from EC45 to EC51 (beginning of heading) as summarized in **Table 1**. Winter wheat was sown on 30 October 2012 and harvested on 23 July 2013 for JB Asano and 2 August 2013 for Tobak. Nitrogen fertilization was conducted on 26 October 2012, 25 March 2013 for both cultivars and on 24 May 2013 for JB Asano and 29 May 2013 for Tobak. Fungi disease and insects were well controlled by spraying fungicide Capalo two times and insecticide Biscaya once during winter wheat growth. The soil used for both pot experiments was classified as sandy loam, pH 6.6, and the nutrient concentrations were 250 mg P_2_O_5_ kg^-1^, 180 mg K_2_O kg^-1^, 3.3 mg S kg^-1^, and 1300 mg N kg^-1^ and organic matter of 2.3%. Soil nutrient analyses were performed according to the regulations of the Association of German Agricultural Analytic and Research Institutes (VDLUFA, 1991).

### Yield, Nitrogen and Protein Concentration

After harvest, grains, straw and glumes were separated, dried and the dry weight of each fraction was determined for each pot. Grains were milled in a Titan laminated mill using a 500 μm sieve (Retsch, Haan, Germany). Nitrogen concentration of each fraction was determined by a CNS elemental analyzer (Flash EA 1112 NCS, Thermo Fisher Scientific, Waltham, MA, USA). Crude protein concentration of wheat flour was calculated by multiplying the N concentration by 5.7.

### Elemental Analysis Isotope Ratio Mass Spectrometry

Grain flour was analyzed by elemental analyzer – stable isotope ratio mass spectrometry (EA-SIRMS) using a DELTA V PLUS Isotope Ratio MS (Thermo Electron Corporation) interfaced with a Flash 2000 Organic Elemental Analyzer (Thermo Fisher Scientific, Waltham, MA, USA) to determine N isotope ratios.

### Extraction and Quantification of Storage Protein Fractions

Extraction of flour storage protein fractions was performed stepwise according to Zörb et al. (2010) with some modifications. First albumins and globulins were extracted from 100 mg wholemeal flour with 1 mL extraction buffer (0.4 mol L^-1^ NaCl, 67 mmol L^-1^ Na_2_HPO_4_, 7.7 mmol L^-1^ KH_2_PO_4_, pH 7.6) using a 2 mL tube. After vortexing shortly the samples were stirred for 20 min at 4°C and centrifuged (16,000 *× g*, 20 min, 4°C). The supernatant was collected as albumin-globulin fraction. This procedure was repeated once. The pellet was then extracted with 500 μL 60% ethanol to obtain the gliadin fraction using the same procedure as described above and repeated twice. The three supernatants were combined as one gliadin fraction. The pellet was then washed with 1 mL of deionized water, stirred for 5 min, and centrifuged (16,000 *× g*, 15 min, 4°C). The supernatant was discarded. Afterward, the glutenin fraction was extracted by adding 1 mL extraction buffer [50% 1-propanol (v/v), 2 mol L^-1^ urea, 1% dithiothreitol (DTT), 0.05 mol L^-1^ Tris-HCl, pH 7.5] to the pellet. Extraction was done by vortexing for one min and stirring for 25 min at 60°C followed by centrifugation (16,000 *× g*, 15 min, 20°C). The supernatant was collected and the extraction repeated once. In the end, all gluten fractions were centrifuged (16,000 *× g*, 25 min, 4°C for gliadin and 20°C for glutenin) and each resulting supernatant was transferred into a new 2 mL tube and stored at -20°C. A subsample (400 μL) of each protein fraction was transferred into a new 2 mL tube and the protein was precipitated by adding 600 μL DTT/acetone solution (50 mmol L^-1^ DTT) and stored at -20°C overnight. After centrifugation (16,000 × *g*, 25 min, 4°C), the supernatant was discarded and the pellet was dissolved by dissolving buffer {8 mol L^-1^ urea (serdolit washed), 2 mol L^-1^ thiourea (serdolit washed), 4% 3-[(3-cholamidopropyl)dimethylammonio]-1-propanesulfonate (CHAPS), 30 mmol L^-1^ DTT, 20 mmol L^-1^ Tris base} and stored at -20°C. The concentration of each protein fraction was determined using 2D Quant-kit (GE-Healthcare, Munich, Germany).

### Sodium Dodecyl Sulfate Polyacrylamide Gel Electrophoresis (SDS-PAGE)

Gliadin and glutenin compositions were determined by SDS-PAGE performed according to the method of Laemmli (1970) with some modifications. Medium sized (18 cm × 16 cm glass plates and 1.5 mm spacers in thickness) gels (12.5% separating gels and 6.3% stacking gels) were casted 1 day before use. Separating gels were prepared by mixing 24 mL deionized water, 18 mL separating gel buffer (1.5 mol L^-1^ Tris base, 0.4% SDS, pH 8.8), 30 mL acrylamide/bis-acrylamide (37.5/1, v/v; Rotiphorese Gel 30), 599.4 μL 10% ammonium persulfate (fresh) and 59.4 μL tetramethylethylenediamine (TEMED) in a beaker and then pipetted between the plates leaving 2 cm space to the top and covered by 2-propanol. After 1 h, 2-propanol was replaced by stacking gel [prepared by mixing 15 mL deionized water, 7.5 mL stacking gel buffer (0.5 mol L^-1^ Tris base, 0.4% SDS, pH 6.8)], 6 mL polyacrylamide, 120 μL 10% ammonium persulfate and 120 μL TEMED in a beaker). A comb was then placed. Protein extracts were mixed with the same volume of sample buffer (10% glycerol, 2.3% SDS, 5% 2-mercaptoethanol, 0.0025% bromophenol blue, 63 mmol L^-1^ Tris-base, pH 6.8) and shaken for 5 min at 95°C and cooled at room temperature. Each well was loaded with sample containing 20 μg protein. A molecular weight standard ‘Rotimark 10-150’ (RotiMark, Roth, Crailsheim, Germany) was loaded on one lane of each gel. Two gels were run simultaneously in the same vertical electrophoretic unit (SE600, Hoefer) at 50 mA and 17°C for around 6 h. The gels were then fixed (40% ethanol, 10% acetic acid), stained (1 tablet ‘PhastGel^TM^ Blue R’ in 1.6 L 10% acetic acid) and destained (10% acetic acid). Gels were digitized by scanning on an image scanner (Epson PerfectionV700) at 300 dpi and 16 bits per pixel.

### Image Analysis

Gel electrophoresis images were analyzed using the Quantity One software (Bio-Rad, Hercules, CA, USA). Bands on each gel were determined and matched with molecular weight markers to calculate the molecular weight of each protein band. The average band intensity and relative quantity of each band were then determined.

### Micro-Baking Test (MBT)

The MBT was performed according to Thanhaeuser et al. (2014) with the modification that wholemeal flour was used. After grinding, wheat flours were stored for at least 14 days before being used for baking. The moisture of each wholemeal flour sample was measured using an infrared moisture analyzer (MA35, Sartorius). Then, for each sample the water absorption and dough development time were determined using a modification of the ICC-Standard method 114/1 by mixing 10 g flour, 0.2 g NaCl, and water at 22°C for 20 min in a micro-farinograph (Brabender, Duisburg, Germany). Afterward, the MBT was performed. Briefly, 10 g flour, 0.7 g fresh yeast (Wieninger, Passau, Germany), 0.2 g NaCl, 0.2 g sucrose, 0.1 g coconut fat, 0.3 mL L-ascorbic acid solution (0.67 g L^-1^) and water were mixed in the micro-farinograph for 4–6 min according to the dough development time. Then the dough was weighed and placed in a proofer for 20 min (30°C, water-saturated atmosphere). After rounding and weighing again, the dough was fermented 35 min on the baking line at 29°C in a water-saturated atmosphere. The dough was then baked for 10 min (three temperature stages: 120°C, 180°C and 250°C).

The bread was weighed immediately and again at 1 h after baking. The bread volume was measured using Volscan Profiler 600 (Stable Micro Systems, Godalming, UK).

### Calculations and Statistical Analysis

Plant N uptake (g N pot^-1^) was calculated according to the following equation:

$$Plant\,N\,\;uptake\,\;(gN\,\;{pot}^{-1})={DW}_{Grain}\times {NC}_{Grain}+{DW}_{straw}\times {NC}_{Straw}+{DW}_{Glume}\times {NC}_{Glume}$$

in which DW_Grain_, DW_Straw_ and DW_Glume_ are dry weight (g pot^-1^) of grains, straws and glumes, respectively; NC_Grain_, NC_Straw_, and NC_Glume_ are N concentration (%) of grains, straw and glumes, respectively.

Nitrogen harvest index (NHI) (%) was calculated according to the following equation:

$$NHI\left(\%\right)=\frac{{DW}_{Grain}\times {NC}_{Grain}}{Plant\,\;N\,\;uptake}\times 100$$

Late N recovery (LNR) (%) was calculated according to the following equation:

LNR(%)=N⁢ excessGrain×NCGrain×DWGrain+15N⁢ excessStraw×NCStraw×DWStraw+15N⁢ excessGlume×NCGlume×DWGlume15N⁢ excessF×F15

in which ^15^N excess_Grain_, ^15^N excess_Straw_, ^15^N excess_Glume_ and ^15^N excess_F_ is the excess of ^15^N in grains, straw, glumes and fertilizer; F is the total N (g) in late N fertilizer.

Late N harvest index (LNHI) (%) was calculated according to the following equation:

$$LNHI(\%)=\frac{{LNR}_{Grain}}{LNR}\times 100$$

in which LNR_Grain_ is the late N recovery (%) in grains.

Data for each parameter was presented as the mean value of five replicates analyzed. The software SPSS 13.0 (Chicago, IL, USA) was used. Significance of treatments within each cultivar was determined at *P* < 0.05 level by analysis of variance (One-way ANOVA) followed by Tukey’s least significant test. Before, a two-way ANOVA was conducted testing treatment and cultivar effects and their interactions in order to get an overview over parameter changes.

## Results

### Grain Yield and N Uptake

Winter wheat grain yield and plant N uptake were increased by late N fertilization for both cultivars and in both experiments through increased N fertilization rate, while split N application had no such effects (**Table 2**). Around 63–68% of the total N uptake was translocated to the kernels. However, N harvest index was mainly enhanced by late nitrate fertilization, except that there was no effect on Tobak in the second experiment, and was also increased with higher N rate on JB Asano in the second experiment (**Table 2**). The recovery of late N fertilizer was higher when applied as nitrate-N compared to urea, except for Tobak in the second experiment (**Table 2**). Comparing to N fertilizer applied at early growth stage of wheat, late N fertilizer was more efficiently used in the kernels, resulting in much higher late N harvest index (80–86%) than N harvest index (63–68%) (**Table 2**).

**Table 2:** Grain yield, plant N uptake, N and late N harvest index and late N recovery as affected by N treatments.

		Grain yield (g pot^-1^)		Plant N uptake (g N pot^-1^)		NHI (%)		Late N harvest index (%)		Late N recovery (%)	
Treatment		Tobak	JB Asano	Tobak	JB Asano	Tobak	JB Asano	Tobak	JB Asano	Tobak	JB Asano
Experiment 1	N1	64.4 b	64.3 b	1.45 b	1.46 b	63.6 b	64.0 b				
	N2	74.5 a	79.5 a	1.81 a	1.84 a	63.7 b	66.0 ab				
	N3	75.5 a	75.0 a	1.80 a	1.84 a	67.6 a	68.0 a	85.2 a	86.0 a	95.6 a	98.1 a
	N4	72.7 a	77.6 a	1.73 a	1.85 a	65.7 ab	66.4 ab	84.5 a	84.2 a	81.6 b	79.6 b

Experiment 2	N5	89.0 b	92.2 b	2.03 b	2.00 b	64.4 a	62.9 c				
	N6	106.1 a	112.6 a	2.47 a	2.52 a	67.1 a	65.4 b				
	N7	111.1 a	113.9 a	2.54 a	2.62 a	67.8 a	67.6 a	83.8 a	80.8 a	89.4 a	95.2 a
	N8	101.5 a	108.0 a	2.45 a	2.48 a	67.1 a	67.2 ab	83.3 a	80.1 a	88.1 a	83.6 b

*Different letters in the same column within the same experiment represent significant differences of mean values at P < 0.05. Treatments N1–N8 represents the corresponding treatments as described in **Table 1**.*

### Protein Concentration and Composition

Protein concentration was mainly increased by higher N rate and further increased by split N application in nitrate-N in JB Asano when total N rate was medium (first experiment) (**Table 3**). However, protein concentration was only increased by late N fertilization in JB Asano when total N rate was further increased (second experiment). Protein yield showed the same trend as grain yield. Furthermore, due to the much higher grain yield in the second experiment and similar protein concentration between both experiments, protein yield in the second experiment was much higher than in the first experiment.

**Table 3:** Protein concentration and composition as affected by N treatments.

		Protein concentration (mg g^-1^ flour)		Protein yield (g pot^-1^)		Gliadin (mg g^-1^ flour)		Glutenin (mg g^-1^ flour)		Gliadin proportion (%)^a^		Glutenin proportion (%)^a^		Gliadin/glutenin		Gluten (mg g^-1^ flour)	
	Treatment	Tobak	JB Asano	Tobak	JB Asano	Tobak	JB Asano	Tobak	JB Asano	Tobak	JB Asano	Tobak	JB Asano	Tobak	JB Asano	Tobak	JB Asano
Experiment 1	N1	81.7 b	82.7 c	5.3 b	5.3 b	12.7 c	12.5 d	19.4 c	18.1 c	15.5 c	15.1 c	23.7 c	21.9 c	0.65 b	0.69 b	32.0 d	30.6 d
	N2	88.4 a	87.0 b	6.6 a	6.9 a	15.8 b	15.5 c	22.7 b	19.8 c	17.9 b	17.8 b	25.7 bc	22.7 bc	0.70 a	0.78 a	38.5 c	35.2 c
	N3	91.9 a	94.9 a	6.9 a	7.1 a	19.4 a	20.4 a	27.3 a	25.7 a	21.1 a	21.5 a	29.8 a	27.1 a	0.71 a	0.79 a	46.7 a	46.2 a
	N4	89.1 a	90.3 b	6.5 a	7.0 a	17.7 a	18.0 b	24.5 b	22.0 b	20.0 a	19.9 a	27.5 ab	24.3 b	0.72 a	0.82 a	42.3 b	39.9 b

Experiment 2	N5	84.0 a	78.0 b	7.5 b	7.2 b	16.8 a	17.6 a	27.4 b	27.8 a	20.1 a	22.5 a	32.6 a	35.6 a	0.62 a	0.64 a	44.2 b	45.3 a
	N6	89.2 a	83.6 ab	9.5 a	9.4 a	18.3 a	18.0 a	31.7 a	29.3 a	20.5 a	21.6 a	35.6 a	35.1 a	0.58 a	0.62 a	50.0 a	47.4 a
	N7	88.5 a	88.6 a	9.8 a	10.1 a	18.6 a	17.1 a	32.0 a	30.0 a	21.1 a	19.3 a	36.3 a	33.9 a	0.58 a	0.58 a	50.6 a	47.1 a
	N8	92.6 a	88.2 a	9.4 a	9.5 a	19.3 a	18.2 a	31.3 a	29.1 a	20.8 a	20.6 a	33.8 a	33.0 a	0.61 a	0.62 a	50.6 a	47.3 a

*^a^Gliadin proportion (%) refers to the percentage of gliadins to total grain protein, and was calculated as gliadin (mg g^-1^ flour)/total grain protein (mg g^-1^ flour) × 100. Glutenin proportion (%) refers to the percentage of glutenins to total grain protein, and was calculated as glutenin (mg g^-1^ flour)/total grain protein (mg g^-1^ flour) × 100. Different letters in the same column within the same experiment represent significant differences of mean values at P < 0.05. Treatments N1–N8 represents the corresponding treatments as described in **Table 1**.*

The effects of late N fertilization on the concentrations and proportions of gluten fractions varied between experiments (**Table 3**). In the first experiment, gliadin concentration, gliadin proportion and gluten concentration were increased by higher N fertilization rate and further increased by split N application. Glutenin concentration was increased by higher N rate and split N application in nitrate-N (Tobak) or just by split N application (JB Asano), whereas glutenin proportion was increased by late N fertilization and split N application in nitrate-N. Although the response of gliadin and glutenin concentration varied among fertilization treatments and cultivars, gliadin to glutenin ratio was only increased through higher N fertilization rate regardless of the timing for both cultivars. Furthermore, nitrate-N had greater effects in increasing concentrations of gluten fractions than urea when applied as late N. Comparing to JB Asano, Tobak had higher glutenin concentration and similar gliadin concentration, thus the gliadin/glutenin ratio was lower in Tobak than in JB Asano.

However, in the second experiment, only glutenin and gluten concentration were increased by late N fertilization through higher N fertilization rate in Tobak (**Table 3**). Late N fertilization had no significant effects on gliadin concentration, glutenin concentration (JB Asano), gliadin and glutenin proportions and their ratio. Besides, there was no difference between N forms for all these parameters.

### Gluten Subunits Pattern

**FIGURE 1:**
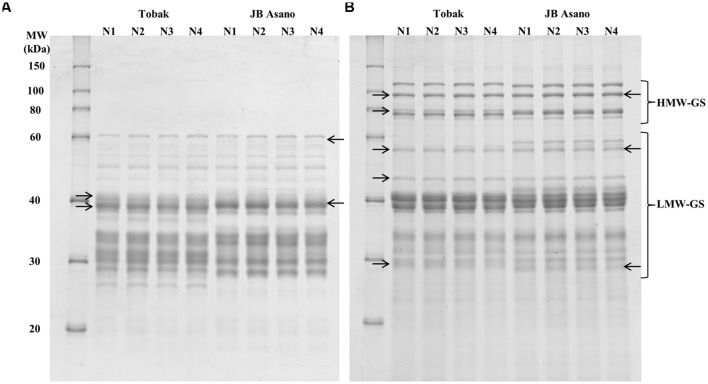
**Effects of N treatments on protein subunits patterns of gliadin (A) and glutenin (B) fractions separated by SDS-PAGE for the two winter wheat cultivars Tobak and JB Asano.** Treatments N1–N4 represents the corresponding treatments as described in **Table 1**. The Right Arrow (→) and the Left Arrow (←) indicate the bands that showed relative changes after different N treatments in Tobak and JB Asano, respectively.

Gliadin and glutenin protein subunits were separated by SDS-PAGE, and the protein band patterns of both cultivars were shown in **Figures 1A,B**. For the gliadin fraction, 14 protein bands were detected and their relative quantities were determined for Tobak and JB Asano, respectively. In the first experiment (**Table 4**), the relative quantities of two γ-gliadin subunits in Tobak (band 6, MW 40.1 kDa and band 7, MW 38.7 kDa) and one γ-gliadin subunit in JB Asano (band 8, MW 39 kDa) were decreased by late N fertilization through the combined effect of higher N rate and split N application, respectively. Besides, one ω-gliadin subunit in JB Asano (band 2, MW 58.4 kDa) was increased with late nitrate fertilization through split N application. However, no change of band relative quantity was found in both cultivars in the second experiment (data not shown).

**Table 4:** Gliadin subunits pattern and their relative quantities (%) of two winter wheat cultivars as affected by N treatments in the first experiment.

Tobak						JB Asano					
Band	MW (kDa)	N1	N2	N3	N4	Band	MW (kDa)	N1	N2	N3	N4
1	60.1	3.6	4.4	4.9	5.1	1	59.9	3.2	3.7	3.8	4.3
2	56.4	1.1	1.3	1.4	1.5	2	58.4	1.2 b	1.2 b	1.9 a	1.3 ab
3	52.9	1.0	1.5	1.5	1.4	3	56.2	1.3	1.7	2.0	2.0
4	49.1	3.0	3.3	3.6	3.7	4	52.8	1.6	1.9	2.2	2.3
5	45.5	1.1	1.1	1.3	1.1	5	48.9	3.1	3.3	3.4	3.6
6	40.1	13.3 a	11.7 ab	11.2 b	11.6 b	6	45.4	1.0	1.2	1.1	1.2
7	38.7	32.8 a	30.3 ab	29.2 b	28.4 b	7	41.8	0.5	0.4	0.5	0.7
8	36.4	1.8	2.3	2.5	2.5	8	39.0	37.3 a	35.3 ab	34.0 b	34.2 b
9	33.1	19.8	19.8	19.6	19.3	9	37.7	6.1	6.6	6.9	6.4
10	30.7	8.9	9.6	9.9	10.0	10	33.5	11.3	11.5	11.6	11.3
11	29.8	5.3	5.9	6.3	7.0	11	32.6	9.8	10.1	10.5	10.2
12	28.5	4.3	4.5	4.1	3.8	12	29.7	11.7	11.4	11.3	11.4
13	27.4	0.3	0.3	0.4	0.4	13	28.1	11.2	10.8	10.3	10.4
14	26.1	3.6	4.0	4.1	4.0	14	25.3	0.7	0.8	0.5	0.7

*Different letters in the same row within the same wheat cultivar represent significant differences of mean values at P < 0.05. The absence of letters in the same row within the same wheat cultivar represents no significant difference of mean values at P < 0.1. Treatments N1–N4 represent the corresponding treatments as described in **Table 1**.*

For the glutenin fraction, 11 and 14 protein bands were determined for Tobak and JB Asano, respectively. In the first experiment (**Table 5**), the relative quantity of four and two glutenin bands in Tobak (band 2, 3, 5, and 6) and JB Asano (band 2 and 6), respectively, were increased by late N fertilization, while one band in Tobak (band 11) and one band in JB Asano (band 14) were decreased by late N fertilization, respectively. Particularly, the relative quantity of the same HMW-GS (subunit 7), band 2 in both Tobak (at *P* < 0.05 level) and JB Asano (at *P* < 0.1 level), was only increased with late N fertilization through split N application. In the second experiment, the relative quantities of two LMW-GS bands were decreased with late N fertilization through higher N rate in JB Asano, while no change was observed in Tobak (data not shown). In terms of HMW-GS to LMW-GS ratio (**Table 5**), JB Asano had higher ratio than Tobak. However, no difference was found between treatments in both cultivars and in both years (data from the second experiment not shown).

**Table 5:** Glutenin subunits pattern and their relative quantities (%) of two winter wheat cultivars as affected by N treatments in the first experiment.

Tobak						JB Asano					
Band	MW (kDa)	N1	N2	N3	N4	Band	MW (kDa)	N1	N2	N3	N4
1	114.5	8.0	7.9	8.5	8.5	1	112.9	9.8	9.9	10.3	10.3
2	96.1	11.9 b	12.0 b	13.1 a	13.0 a	2	96.9	13.5 b	13.5 b	14.3 a	14.2 a
3	79.1	2.9 b	3.1 ab	3.1 ab	3.3 a	3	77.7	14.2	14.6	14.5	14.4
4	76.2	10.6	11.0	11.2	11.5	4	58.6	3.7	3.9	3.8	3.7
5	55.8	3.1 b	3.9 a	3.8 a	3.8 a	5	55.7	5.4	5.8	5.6	5.1
6	46.2	2.4 b	2.7 ab	2.9 a	2.9 a	6	46.3	2.9 b	3.4 ab	3.7 a	3.6 a
7	40.5	24.2	23.6	24.2	24.4	7	43.1	0.6	0.4	0.5	0.5
8	38.8	21.7	21.0	21.4	20.6	8	40.5	18.5	18.2	19.0	19.4
9	33.6	6.3	6.3	5.3	5.7	9	39.1	10.6	10.8	11.3	11.5
10	31.4	1.8	1.9	2.0	2.2	10	38.2	2.2	2.7	2.8	2.6
11	29.2	7.1 a	6.6 a	4.6 b	4.2 b	11	33.7	8.9	8.6	7.6	7.9
						12	31.2	2.1	2.2	2.2	2.2
						13	29.9	3.0	2.7	2.2	2.5
						14	28.6	4.6 a	3.2 ab	2.2 b	2.1 b
HMW-GS/LMW-GS		0.50	0.52	0.56	0.57	HMW-GS/LMW-GS		0.60	0.61	0.64	0.64

*Different letters in the same row within the same cultivar represent significant differences of mean values at P < 0.05. For band 2 in JB Asano, different letters in the same row represent significant differences of mean values at P < 0.1. The absence of letters in the same row within the same wheat cultivar represents no significant difference of mean values at P < 0.1. Treatments N1–N4 represents the corresponding treatments as described in **Table 1**.*

### Baking Quality

**FIGURE 2:**
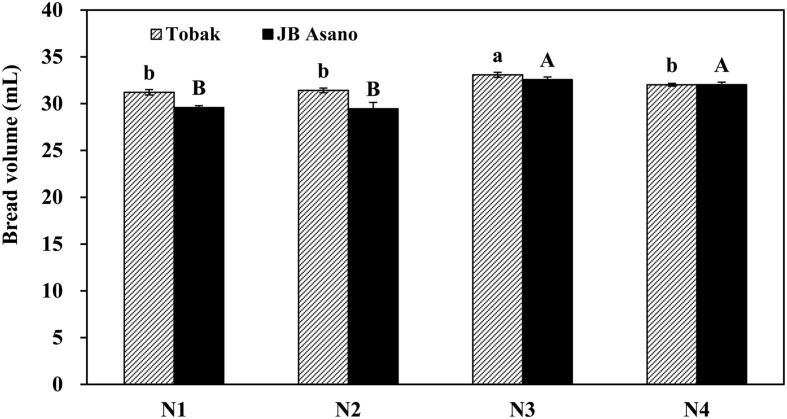
**Bread volume of two winter wheat cultivars as affected by N treatments in the first experiment.** Error bars represent ± standard errors of five independent pot replicates. Statistical significance (*P* < 0.05) is indicated by small letters for Tobak and capitals for JB Asano. Treatments N1–N4 represents the corresponding treatments as described in **Table 1**.

Micro-baking tests were performed only for the first experiment since differences in gluten concentration and composition were mainly observed in the first experiment. For both wheat cultivars, baking quality of wheat flour expressed as bread volume was improved by late N fertilization through split N application (**Figure 2**). Besides, nitrate-N was better than urea in improving flour baking quality when applied as late N, since bread volume of Tobak was only increased by late nitrate fertilization. Furthermore, the positive effect of late N fertilization on JB Asano was greater than on Tobak. JB Asano had a lower bread volume than Tobak when N fertilizer was only applied in two instead of three doses, however, this difference no longer existed after a late N application since bread volume of JB Asano increased more (from 29.5 to 32.3 mL, increased by 9.4%) by late N fertilization than Tobak (from 31.3 to 32.6 mL, increased by 3.9%).

## Discussion

### Protein and Baking Quality of Wheat Flour as Influenced by Late N Fertilization

In our study, late N fertilization increased bread volume of wheat flour through the enhanced grain protein concentration, gliadin and glutenin concentrations as well as their proportions, and relative quantity of certain HMW-GS in the first experiment when the total N fertilization rate was low to medium (from 1.5 to 2 g N pot^-1^). However, the effects of late N fertilization were less obvious under higher N fertilization conditions in the second experiment (from 2 to 2.5 g N pot^-1^). Since the total N fertilization rate was not high in the first experiment, it could be speculated that the N fertilizer applied at early stages was all taken up by the plant before anthesis, while there was still plant N uptake in the split N application treatment. Therefore, this lately uptake of N was more directly used for grain protein synthesis, affecting not only protein quantity but also the N partitioning in the final grain (further discussed below). However, although 2.5 g N pot^-1^ was given at early growth stages in the second experiment (treatment N6), it might not be taken up completely before anthesis. Therefore there might still have been plant N uptake in both treatments N6 and late N fertilization treatments after anthesis. Consequently, the effect of split N application was not observed in the second experiment. Based on these results, the discussion of the present study was mainly focused on the first experiment at the N fertilization rate between 1.5 and 2 g N pot^-1^.

In agreement with other studies (Wieser and Seilmeier, 1998; Blandino et al., 2015), our results confirmed that the late N fertilization was an effective measure in increasing grain protein concentration (**Table 3**). The results also clearly indicated that this effect was mainly due to the increased N fertilization rate rather than split N application since the plant N uptake (**Table 2**) was only increased by higher N fertilization rate, thus more N was available for grain protein synthesis. The fact that grain protein concentration was increased by higher N fertilization rate was already described in many studies (Wieser and Seilmeier, 1998; Garrido-Lestache et al., 2005). Therefore, with respect to the inconsistent results describing the effect of split N application on grain protein concentration (Garrido-Lestache et al., 2004, 2005; Fuertes-Mendizábal et al., 2010; Schulz et al., 2015), it might be explained by variations in plant N uptake. Since different climatic conditions varying in total precipitation or its distribution would have affected the fertilizer N availability in the soil and thus plant N uptake, which led to the same variations as caused by different N fertilization rate.

With the increase in protein concentration, the gliadin and glutenin concentrations as well as their ratio were also increased (**Table 3**). This was in agreement with other studies (Wieser and Seilmeier, 1998; Triboï et al., 2003; Martre et al., 2006). Besides, in agreement with Jia et al. (1996) increasing N supply led to an increase of gliadin proportion, while that of glutenins was not changed. However, split N application further increased gliadin and glutenin concentrations and proportions at the same N fertilization rate (**Table 3**). In contrast to literatures describing that the process of N partitioning in the grain was not significantly altered by environmental conditions and depended mostly on the grain protein content (Triboï et al., 2003), our results showed that split N application affected N partitioning in the grain, increasing mainly gliadin as well as glutenin proportions (**Table 3**). One possible explanation for this split N application effect may due to the two sources of amino acids exported to the phloem for protein synthesis in the grain. One export pool that supplies amino acids to the phloem is fed by the recently reduced nitrate-N and depends on the N uptake by the plant. A second storage pool is where amino acids accumulate when N is not limiting, and are slowly released after the export pool is depleted (Barneix, 2007). The amino acids more readily available for export to the phloem are those that have been synthesized from the reduction of the recently absorbed nitrate-N (Yoneyama and Takeba, 1984). It can be speculated from our results that the corresponding quantitative reduction of non-storage protein fractions at the expense of increased gliadins and glutenins existed, although some studies described that the quantity of structural/metabolic proteins were scarcely influenced by N nutrition (Triboï et al., 2003). Nevertheless, the proportional changes of gliadin and glutenin fractions by split N application in our study were relatively small compared to the broad ranges that have been reported and used for modeling (Martre et al., 2006). However, it had a decisive effect on baking quality (**Figure 2**), and therefore needs to be acknowledged.

Bread wheat cultivars express between three and five HMW-GS. Although HMW-GS account for only up to 12% of entire grain protein in wheat, 45–70% of the variation in baking quality was supposed to be determined by these glutenin subunits within European wheats (Shewry et al., 2001a). Both increases (Wieser and Seilmeier, 1998) and decreases (Pechanek et al., 1997) in the proportions of HMW-GS have been reported in response to N application. However, in contrast to literatures describing that the relative proportions of each HMW-GS remained constant regardless of the increment of N fertilization rate or its splitting (Fuertes-Mendizábal et al., 2010), our results showed that the relative quantities of certain HMW-GS were increased by late N fertilization (**Table 5**). Particularly, the relative quantity of the same HMW-GS (subunit 7) in both cultivars was increased by late N fertilization only through the effect of split N application. Generally, the responses of protein subunits to N fertilization are related to the proportion of the sulfur (S)-containing amino acids in the different gluten protein classes. The proportions of low S-proteins (ω-gliadins) and low to medium S-proteins (such as HMW-GS) increased and those high S-proteins (such as LMW-GS) remained the same or decreased with N fertilization (Daniel and Triboi, 2000; Shewry et al., 2001b; Hurkman et al., 2013). Therefore, it can be speculated that split N application might promote the relative availability of N compared to S, resulting in the proportional increases of certain HMW-GS.

Based on our results from both experiments, it is suggested that the late N fertilization, especially split N application can be crucial in improving baking quality of wheat flours. Although these effects might be highly affected or even hidden by environmental factors such as total N fertilization rate and variations from years and locations, it should not be easily considered to abandon the late N application in order to produce high quality wheat.

### Protein and Baking Quality of Wheat Flour as Affected by N Forms

In the present study, the late application of N was taken up in nitrate-N form in treatment N3 and ammonium-N form in treatment N4. It was hypothesized that the process of amino acid synthesis from ammonium-fed wheat would be enhanced, thus promoting protein accumulation in the final grain. However, results from our study showed that nitrate-N had greater effects than ammonium-N in improving grain protein quality such as gliadin and glutenin concentrations and proportions (**Table 3**) and baking quality (**Figure 2**). The differences between nitrate-N and ammonium-N on grain protein composition and baking quality mainly resulted from the uptake of late N fertilizer, since the LNR from late nitrate application was significantly higher than that from late urea application (**Table 2**). However, our results were in contrast to a recent study by Fuertes-Mendizábal et al. (2013) who reported that ammonium-N as sole N source improved wheat grain quality compared to a nitrate nutrition when split into two and especially three doses. In that study, the N source (NO_3_^-^ or NH_4_^+^) was varied during the whole growth period of wheat. The different results to our study might therefore arise from the fact that no N storage in vacuoles occurred in ammonium-fed plants, therefore the second and third application of ammonium-N was more efficiently used for plant growth and protein synthesis than in nitrate-fed plants, as we originally expected in our hypothesis. Another possible explanation was that the plant N uptake in nitrate-fed plants might be lower than in ammonium-fed plants due to N loss such as nitrate-N leaching. However, the plant N uptake was not determined in that study.

### Cultivar Differences in Baking Quality as Affected by Late N Fertilization

In terms of cultivar responses to late N fertilization, the improvement of loaf volume in JB Asano was greater than that in Tobak (**Figure 2**). Based on the quality parameters according to the German Federal Office of Plant Varieties, JB Asano is a cultivar that usually contains higher protein concentrations at optimum baking quality as compared to Tobak. It can be assumed that JB Asano might be more dependent on grain protein concentration to achieve a certain baking quality. Therefore, the bread volume of JB Asano was eventually increased to a higher extent with the improvement in protein concentration and composition compared to Tobak. These results indicated that measures for enhancing grain protein concentration and composition might be less necessary for cultivars such as Tobak in order to get optimum baking quality. A possible reason for this might be the mostly higher glutenin proportions in the flour of Tobak compared to JB Asano (**Table 3**).

### Meaningful Parameters in Evaluating Wheat Flour Baking Quality

Proteins (mainly protein concentration) are generally recognized to be positively correlated to the bread-making quality within one cultivar (Delcour et al., 2012). However, Johansson and Svensson (1998) investigated two spring and two winter wheat cultivars during 21 years and found that although a positive relationship existed between grain protein concentration and bread volume, the coefficient of correlation was lower than 0.57, and grain protein concentration could only explain 19% of the variation during the investigated years. In the present study, we also demonstrated that protein concentration was not an appropriate parameter in evaluating baking quality of wheat flour since higher N rate increased protein concentration in both wheat cultivars while did not lead to higher bread volume (**Table 3**; **Figure 2**). It has already been reported that although HMW-GS are minor components of gluten in quantitative terms, they are key factors that determine the gluten strength to a great extent and improve baking quality (Shewry et al., 2001a). Besides, it was demonstrated that among HMW-GS, x-type subunits had higher contributions to bread-making quality than y-type subunits (Wieser and Kieffer, 2001). In the present study, x-type HMW-GS subunit 7 present in both wheat cultivars was the most abundant HMW-GS in Tobak (accounting for 36% of the HMW-GS) and second abundant in JB Asano (accounting for 36% of the HMW-GS) (**Table 5**). Consequently, the relative change of this subunit may exert great influences on baking quality, as was observed in our study.

## Conclusion

Late N fertilization has a significant effect in improving baking quality (loaf volume) of wheat flour. This quality improvement is only due to the effect of split N application. Split N application can change the N partitioning in wheat grain by increasing gliadin and glutenin proportions, which is decisive for baking quality. Therefore, although this effect of split N application might be blurred by total N rate effect or hidden by other environmental factors, the practice of split N application should not be abandoned without considering these factors. Grain protein concentration alone is insufficient for evaluating late N effects on baking quality of wheat flour. The proportions of glutenin and HMW-GS seem to be better correlated with baking quality in these cases.

Our results suggest that a late N dose is effective in improving wheat quality because of the N splitting effect while the increase in total N seems less effective. This offers the potential to cut down N fertilization rates in wheat production systems, when the right timing for the N doses is considered. However, this effect cannot be monitored by grain protein concentrations only, so that better parameters to quantify wheat quality have to be adopted in future.

## Author Contributions

CX and AR conducted experiments and analyses with supervision of GSE; RS performed baking tests supervised by PK; CX and GSE wrote manuscript; KHM proposed research.

## Conflict of Interest Statement

The authors declare that the research was conducted in the absence of any commercial or financial relationships that could be construed as a potential conflict of interest.
